# Creation of clinical algorithms for decision-making in oncology: an example with dose prescription in radiation oncology

**DOI:** 10.1186/s12911-021-01568-w

**Published:** 2021-07-12

**Authors:** Fabio Dennstädt, Theresa Treffers, Thomas Iseli, Cédric Panje, Paul Martin Putora

**Affiliations:** 1 Department of Radiation Oncology, Kantonsspital St. Gallen, Rorschacherstrasse 95, 9000 St. Gallen, Switzerland; 2 Seeburg Castle University, Seekirchen am Wallersee, Austria; 3 TUM School of Management, Technical University of Munich, Munich, Germany; 4 Department of Radiation Oncology, University of Berne, Berne, Switzerland

**Keywords:** Decision-making, Clinical algorithm, Decision strategy, Radiation oncology, Dose prescription, Bone metastases

## Abstract

In oncology, decision-making in individual situations is often very complex. To deal with such complexity, people tend to reduce it by relying on their initial intuition. The downside of this intuitive, subjective way of decision-making is that it is prone to cognitive and emotional biases such as overestimating the quality of its judgements or being influenced by one’s current mood. Hence, clinical predictions based on intuition often turn out to be wrong and to be outperformed by statistical predictions. Structuring and objectivizing oncological decision-making may thus overcome some of these issues and have advantages such as avoidance of unwarranted clinical practice variance or error-prevention. Even for uncertain situations with limited medical evidence available or controversies about the best treatment option, structured decision-making approaches like clinical algorithms could outperform intuitive decision-making. However, the idea of such algorithms is not to prescribe the clinician which decision to make nor to abolish medical judgement, but to support physicians in making decisions in a systematic and structured manner. An example for a use-case scenario where such an approach may be feasible is the selection of treatment dose in radiation oncology. In this paper, we will describe how a clinical algorithm for selection of a fractionation scheme for palliative irradiation of bone metastases can be created. We explain which steps in the creation process of a clinical algorithm for supporting decision-making need to be  performed and which challenges and limitations have to be considered.

## Background

Most of the work done by physicians in oncology relies to some level on decision-making. This includes definition of indication, evaluation of the oncological situation and available treatment options, as well as reacting to treatment-related side effects and complications. In individual clinical situations evidence regarding which decision is best is often lacking. While guidelines often provide just a vague orientation, decision-making is mostly done in a more or less intuitive and open way, based on experience, personal opinions and emotions [1, 2]. While being highly flexible, intuitive decision-making can be problematic, as it tends to be inconsistent and is prone to biases [3]. While the growing oncological knowledge is becoming more and more complex, physicians are challenged with difficult decisions in situations of big uncertainties. Due to that, support of clinical decision-making, e.g. via Clinical Decision Support Systems (CDSS), is a field of emerging interest, particularly in oncology [4] and radiation oncology [5]. Structuring of decision-making, even in situations of uncertainties and lacking evidence, may hold great advantages, which can be explained by decision-making theory. Instead of making complex oncological decisions in an unstructured manner, we propose the usage of structured approaches for decision-making, such as clinical algorithms. In contrast to classical guidelines, the idea of clinical algorithms is to provide a clear decisional recommendation for any given situation, depending on clear and assessable decision criteria. Obviously, these recommendations are not always the one and only correct solution for a decisional problem and still need to be evaluated by physicians. Yet, they provide valuable orientation and may lead to more consistency and transparency in the decision-making process.

We are going to methodologically explain how such clinical algorithms for supporting decision-making in oncology can be created and what the challenges and possible solutions for a useful application are. We are going to create a simple algorithm for the palliative irradiation of bone metastases to provide a clinical scenario.

Our aim is to examine the concept of structured decision-making in radiotherapeutic dose selection as well as problems and possible solutions associated with it. We want to provide a framework for how a structured approach in the form of a clinical algorithm can be created and demonstrate what the necessary steps in the creation process are.

## Decision-making theory and medicine

### System-1 and system-2 thinking

Decision-making is a process that is composed of three parts and results in a decision [6]. First, the decision-maker considers all possible courses of action. Second, the decision-maker forms expectations concerning future events and outcomes. Finally, the consequences associated with the possible outcomes are assessed based on a range of outcome parameters (e.g., current goals, personal preferences) and the decision is made as a result of this assessment.

**Fig. 1:**
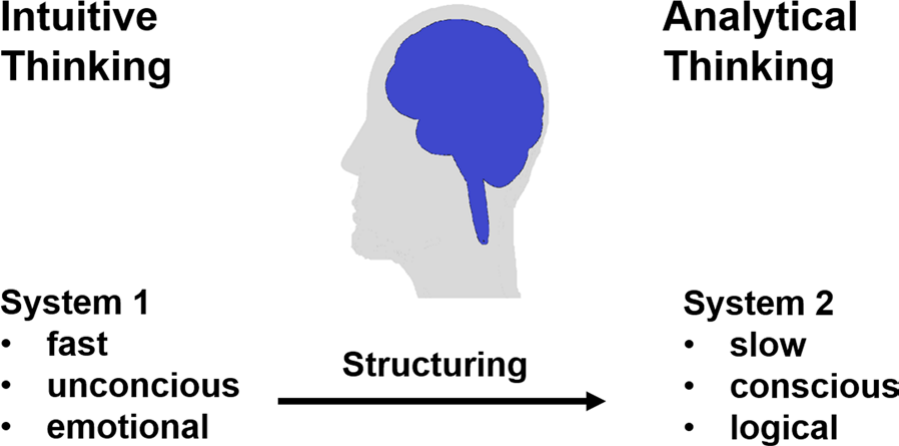
The two systems of information processing

During this decision-making process, people use two different processing systems [7]**.** System 1 is based on intuitive thinking and associated with fast, automatic, emotional and unconscious information processing; system 2 is based on analytic thinking and associated with slow, effortful, systematic and structured information processing (Fig. 1). While system 1 helps us to make fast and automatic decisions in daily life, it is not able to perform complex calculations and statistical analysis and is prone to cognitive and emotional biases. For important and complex decisions, system 2 is superior as it relies on structured and analytical information processing. The downside of system 2 is that it is slow and effortful.

In clinical decision-making we usually use both systems. While evaluation of scientific studies and statistical data is fundamental to modern evidence-based medicine, intuition is a main part of clinical reasoning [1]. However, system 1 is open to (un)conscious biases such as emotions, overconfidence, framing effects or stereotypes, and these biases may lead to suboptimal patient care [8].

An example for the influence of such biases was shown by McNeil et al. who compared clinical decision-making for the treatment of lung cancer in a psychological study [9]. Participating physicians were shown data summarizing the outcome of the two treatment modalities surgery and radiation therapy. The way in which the data was presented had a considerable impact on the decision-making. The two formulations “90% chance of surviving” and “10% chance of dying” are semantically identical and it should not matter which one is used to describe a treatment outcome. Yet, the first has a positive emotional association (surviving), while the latter has a negative one (dying). As a result, system-1-thinking may recommend or refuse a decision, depending on the emotional context of the situation, choosing 90% survival over 10% death.

Social psychology research has shown that emotional biases can be reduced or eliminated from personal judgments when making people aware of the irrelevant source of their emotion for the decision at hand [10]. Furthermore, unconscious biases can be eliminated by introducing structures and processes that can minimize the effect of biases on the decision-making process. Hence, in an effort to limit the impact of system 1 and instead promote system 2 for decision-making and come to more objective decisions, it is possible to introduce and establish predefined structures and processes.

### Clinical versus statistical predictions

With the goal to make high-quality decisions, it is important to make accurate predictions about the future. Decisions are made depending on which choice is predicted to result in the best outcome. In 1954 the psychologist Paul Meehl published his work about the comparison between clinical and statistical prediction [11]. In several studies he demonstrated that statistical approaches using formal algorithms typically  have a better predictive accuracy than intuitive estimations commonly used by clinicians. These initial findings have been confirmed over decades in different settings [12].

An oncological example for this is the survival estimation of palliative cancer patients. It has been repeatedly shown that clinicians’ intuitive predictions are mostly inaccurate and tend to be too optimistic [13]. As a result, different systematic strategies for survival time estimation from prognostic tools [14] and scores to machine learning models [15] have been developed, which is a field of ongoing research. Such systematic approaches can be continuously improved and can lead to more accurate results than intuitive  estimates.

Nevertheless, intuitive system-1-thinking is very powerful and valuable. Without much effort, it can make decisions even in highly complex and uncertain situations. System-1-thinking may be biased leading to results that are far from perfect, but in daily life it is better to make suboptimal decisions with ease than doing effortful calculations every time a new decision has to be made. Good intuition is based on unconscious associations that evolve with experience and learning. While this may lead to good results for short-term predictions, statistical approaches are superior in situations without a direct feedback of whether a made prediction was correct or not [16]. Without immediate feedback, there is barely any learning effect and intuitive predictions do not improve. As a result, the development of experience-based expertise is limited. At the same time, experts tend to overestimate the quality of their own predictions based on intuition instead of statistical knowledge. For long-term predictions, statistical methods and algorithms have a better predictive value and lead to less biased decisions.

As it appears not as effective to fight unconscious biases with conscious effort, it is reasonable to use highly structured approaches such as clinical algorithms to support the clinical decision-making process.

## The clinical algorithm

“Algorithm” is a quite general term, that is used to describe different concepts with often overlapping definitions. These concepts range from simple flowcharts and step-by-step protocols to complex computer-driven methods, including machine-learning techniques and artificial intelligence systems.

In this work we will focus on the creation and discussion of clinical algorithms, which may be described as “stepwise procedure[s] for making decisions about the diagnosis and treatment of a clinical problem” [17] to “improve the delivery of medical care” [18].

While the clinical algorithm can be implemented into software (e.g. via a CDSS), the algorithm itself is detached from IT implementations and is often depicted via flowcharts, decision trees or protocols [17]. It is a rather simple set of instructions, which can be straightforwardly executed by humans.

Regarding this concept, many different terms like “clinical protocols”, “clinical practice guidelines”, “medical algorithms”, “care algorithms”, etc. with overlapping meanings are often used to describe the same thing [19]. In order to avoid confusion, we will be using the term clinical algorithm for this kind of approach.

They should not be confused with other algorithms, which may also be used in medicine, such as IT-based computer algorithms or decision tree learning algorithms applied in healthcare software development and data mining. While these kinds of approaches can just as well be used for making predictions and supporting decision-making (e.g. for assessing cancer drug efficacy in chemotherapy [20]), they are different from clinical algorithms.

Clinical algorithms in the form of clinical standards, practical guidelines or standard operating procedures (SOPs) play an important role in medicine. In certain fields like in emergency medicine, algorithms like the Advanced Cardiac Life Support (ACLS) or the Advanced Trauma Life Support (ATLS) algorithm provide a standard for patient care that is  widely used among different facilities [21]. Another famous example is the Goldman algorithm developed in 1982 for identification of myocardial infarction in patients presenting with chest pain in emergency departments [22]. Adherence to this simple algorithm led to more specific and equally sensitive results at lower costs compared to regular physicians’ clinical decision-making in a prospective multi-center analysis [23]. Examples like these show the vast potential of using  clinical algorithms for  medical decision-making.

One of the few publications focusing on clinical algorithms per se was provided by Green and Defoe, in which they mention time saving, error prevention, consistency and standardization, as well as learning opportunities as some of the advantages of clinical algorithms [24].

## Creation of a clinical algorithm: dose selection for palliative radiotherapy of bone metastases

Our aim is to illustrate how a useful clinical algorithm for decision-making in oncology can be created.

An often used method for developing these kind of algorithms was described by Horabin and Lewis [25], consisting of the following 7 steps:

1. Identifying performance requirement
2. Defining the problem
3. Defining who will solve the problem
4. Defining how to use the algorithm (teaching, guiding care or both)
5. Developing a first draft
6. Testing and revision until graphic acceptability is achieved
7. Testing and revision until learning or performance effectiveness is achieved

**Fig. 2:**
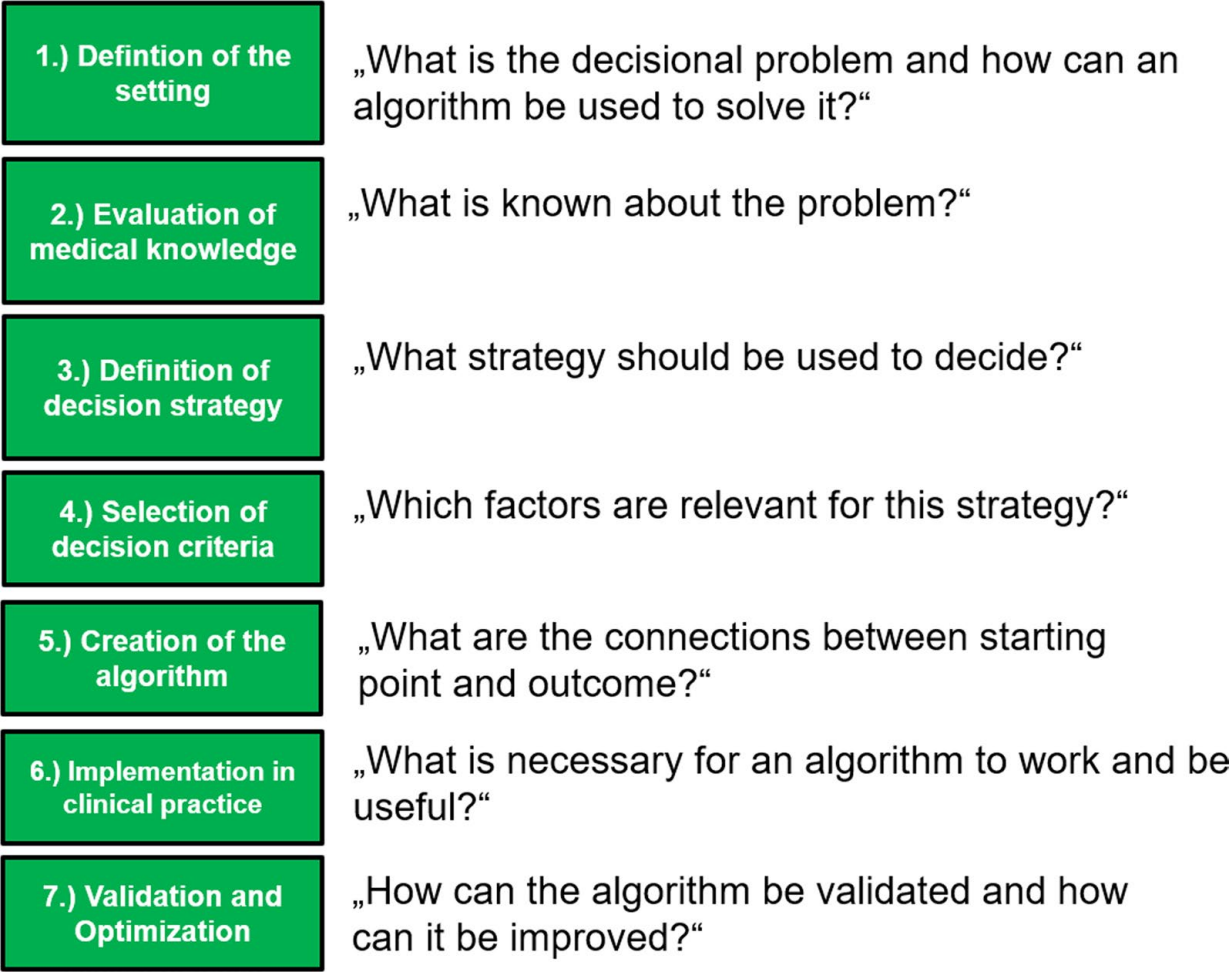
Necessary steps for the creation and application of a clinical algorithm for supporting decision-making in oncology together with the key questions that need to be answered in every step

Since this method is quite general for creating algorithms, we will use a similar approach, analogously consisting of 7 steps, that can be applied for the creation of clinical algorithms for supporting decision-making in oncology. Figure 2 shows a schematic illustration of this method and the main questions that need to be answered in the creation process. We will demonstrate how this framework can be used to create a dose selection algorithm for the palliative irradiation of bone metastases.

### Step 1: Definition of the setting

First, it is important to clearly define the setting, for which a clinical algorithm should be created. This involves a clear definition of the decisional problem, defining who will use the algorithm and defining what purpose the algorithm has (analogous to the steps 2–4 of Horabin and Lewis [25]).

**Fig. 3:**
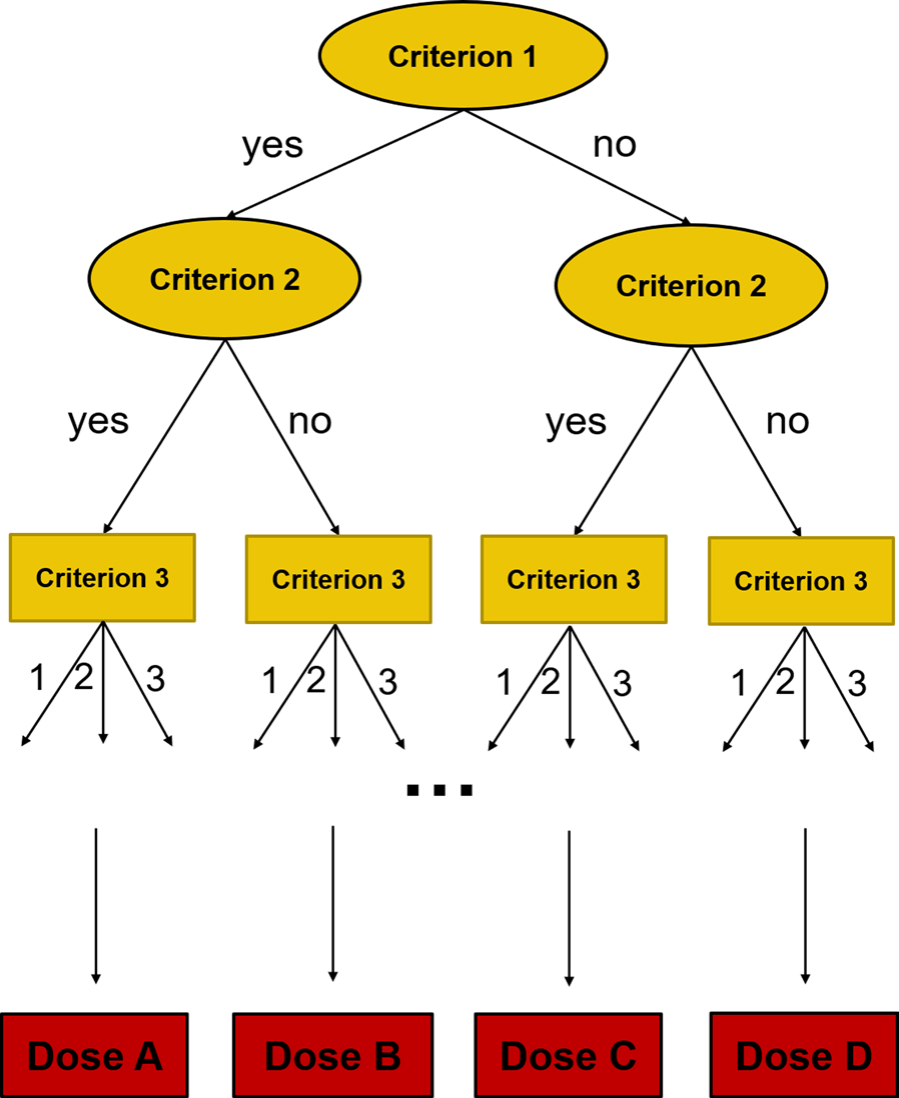
Visualization of structured decision-making in form of a decision tree. Input parameters are used as decision criteria, while output parameters are the decisional options (dose)

The definition of dose prescription (overall dose and fractionation) for a target volume is a typical medical decision in radiation oncology. Uncertainty regarding which option fits an individual patients’ situation best is a common problem. A decision-making analysis of 23 different facilities within the same healthcare setting (Switzerland) on the primary radiotherapy of prostate cancer demonstrated considerable variations regarding dose prescription and recommendations for androgen deprivation therapy [26]. While there is high variability and missing consensus, the choice of radiation dose can be perceived as an isolated decisional problem. This problem is represented within a  defined scope with clear input (decision criteria) and output (choice of dose). Due to this circumstance, this decisional problem may be eligible for solving with a structured approach. Figure 3 shows the concept of using a clinical algorithm for dose prescription based on decision criteria.

As our clinical example, we will create a clinical algorithm for the dose selection in palliative irradiation of bone metastases. It is intended to be used by physicians and the purpose of the algorithm is guidance of decision-making.

### Step 2: Evaluation of medical knowledge: palliative radiation of bone metastases and the question of the optimal dose prescription

After definition of the setting in which a clinical algorithm should be created, one should summarize what is known about the decisional problem. It is especially important to be aware of the uncertainties and controversies about the subject. For dose selection in palliative irradiation of bone metastases we briefly summarize what is known about this issue.

As the majority of patients with metastatic cancer develop bone metastases, palliative treatment of bone metastases is a common indication for radiotherapy [27], being an effective and cost-efficient treatment option [28]. The therapy goals may include analgesia, re-calcification, prevention/treatment of spinal cord compression or improvement of local control after surgery. Furthermore, re-irradiation of bone metastases due to local tumor relapse or insufficient treatment response may occur [29]. Clinically relevant complications of bone metastases include bone fracture, nerve damage or bone marrow suppression.

Several accepted fractionation regimes (e.g., 1 × 8 Gy, 5 × 4 Gy or 10 × 3 Gy) with different advantages and problems are being used in daily clinical practice. For analgetic irradiation, randomized controlled trials comparing single-fractionation (SF, 1 × 8 Gy) and multiple-fractionation (MF, 5 × 4 Gy or 10 × 3 Gy) schemes show equivalent pain relief. While SF offers higher patient convenience with shorter treatment time, rates of retreatment are significantly higher (20% for SF vs. 8% for MF) [30]. Furthermore, for the irradiation of osteolytic bone metastases MF schemes lead to higher rates of recalcification [31].

Regarding side effects and complications, it is not clear, whether or not there is a difference between SF and MF. While some smaller studies showed an association between SF and increased risk of pathologic fracture [32], the majority of research found no difference [30].

As it often remains controversial, which fractionation scheme is best for an individual patient, the American Society for Radiation Oncology (ASTRO), published an evidence-based guideline on palliative radiation therapy for bone metastases [33]. They state that a SF of 1 × 8 Gy may be particularly convenient for patients with limited life expectancy. Furthermore, as there is level I evidence showing equivalency of SF and MF regarding pain relief, it is proposed to use 1 × 8 Gy for the treatment of most uncomplicated bone metastases [34]. Yet, the majority of radiation oncologists continue to prescribe MF schemes in situations that can be considered “uncomplicated” [35]. Multiple reasons such as reimbursement issues or lacking knowledge have been postulated for this discrepancy [36, 37]. It has been claimed that there is a reluctance to practice evidence-based medicine by prescribing unwarranted MF, leading to unnecessary hospital visits and costs [38].

Yet, for complicated bone metastases on the other hand, it may be reasonable to apply higher doses, using MF schemes. Such complicated situations often include metastases in the spine that can lead to nerve compression. For these situations it has been proposed to use SF for patients with poor prognosis and MF for patients with a longer estimated survival [39, 40]. As the application of higher doses is associated with improved local control [41], dose escalation beyond 10 × 3 Gy may be beneficial for selected patients [42], which is currently being investigated in a prospective multicenter study [43].

The Dutch national guideline “Spinal metastases” provides recommendations on which radiation scheme to choose in an individual situation [44]. They state that reasons to perform a more intensive treatment of ≥ 10 × 3 Gy are situations with a limited number of metastases (oligometastatic disease), as well as in patients with a good prognosis. On the other hand, for patients with a life expectancy of less than one year, it is recommended to use a SF scheme of 1 × 8 Gy.

### Step 3: Definition of decision strategy: single fraction wherever possible, multiple fractions wherever necessary

The creation of a clinical algorithm requires the definition of a decision strategy. As patients with bone metastases and planned radiotherapy treatment have a limited prognosis, the determination of dose intensity is not only based on tumor control probability. Avoiding unwarranted hospital visits and patient convenience stand in contrast to maximizing tumor control by intensification of treatment.

Defining the decision strategy is a crucial step as it determines what kind of clinical algorithm is going to be created. Yet, since there is not enough evidence to clarify which is the best decision in every situation, it is also a step without a clear answer, which is why the strategy has to be selected by choice of the creator of the clinical algorithm. One strategy might be to always choose SF and repeat (and maybe intensify) the therapy in case of relapse. Another strategy might be to liberally choose MF schemes in order to minimize the risk of treatment failure. Obviously, these different strategies lead to different clinical algorithms and have eminent implications on clinical practice.

For our clinical scenario we are going to create an algorithm in accordance with the recommendations given by the aforementioned ASTRO guideline, as well as with the Dutch guideline on spinal metastases. The basic principle of our strategy is to choose SF wherever possible and MF where necessary. On this basis we define the following objectives for the creation of the clinical algorithm:

- Identify relevant decision criteria as well as commonly accepted treatment options in the setting of palliative radiotherapy for bone metastases
- In uncomplicated bone metastases a SF of 1 × 8 Gy should be used
- In complicated bone metastases MF schemes should be evaluated depending on the prognosis and the tumor load

### Step 4: Selection of decision criteria: complexity of the situation and prognosis

An individual oncological problem can contain many different parameters. In clinical practice, a multitude of criteria, patient and caretaker-based factors, as well as environmental factors (e.g. healthcare system, reimbursement) may influence decision-making. Apparently, not all factors can be integrated into a structured approach. Therefore, it is important to identify and select the most relevant criteria.

Besides relevance, the possibility to assess criteria is a prerequisite for implementing them in algorithms. Criteria may present at different levels of objectivity and inter-observer variability. While some may be easily measurable, such as age, others might need a surrogate, such as fitness. These need to be composed of different measurable or objective parameters to guarantee reproducibility.

### Criteria for the dose selection algorithm

Considering the objectives we defined for the algorithm, we can focus on three relevant criteria:

- Whether the metastasis is complicated or uncomplicated
- Tumor load
- Life expectancy

**Fig. 4:**
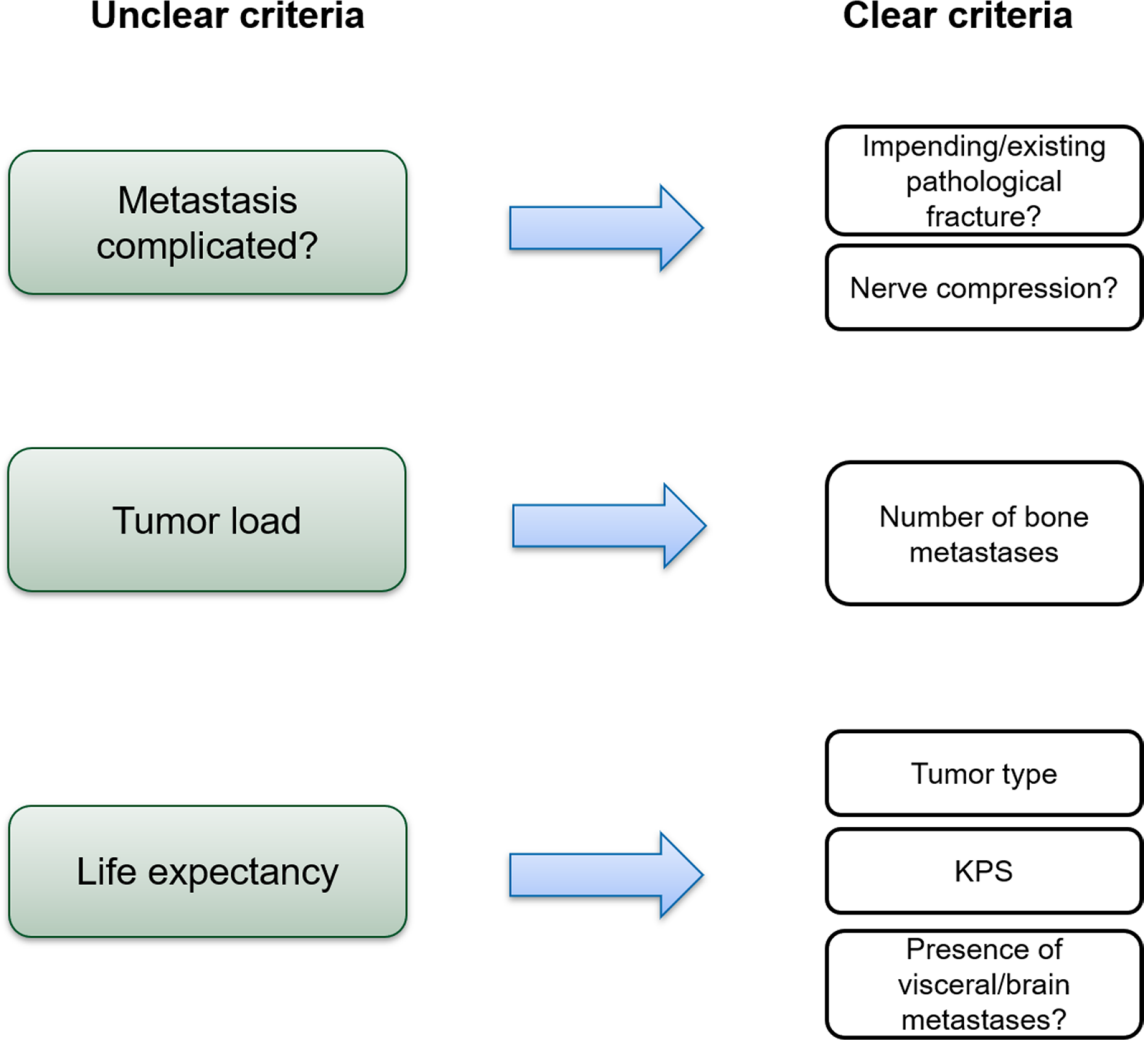
Replacement of unclear criteria with more assessable criteria. As unclear criteria cannot be objectively assessed and are therefore not suitable for implementation into an algorithm, other criteria must be used instead

As the mentioned criteria are more or less unclear and not directly assessable, they have to be addressed using other, more determinable parameters (Fig. 4). Analyzing the studies showing equivalency of MF and SF schemes regarding pain relief, Cheon et al. identified the relevant differences between “complicated” and “uncomplicated” bone metastases [45]. According to this publication, “uncomplicated” bone metastases are not associated with an impending/existing pathological fracture and are not associated with spinal cord or cauda equina compression. We may therefore use these two factors as criteria for our clinical algorithm.

In alignment with the ASTRO guideline, 1 × 8 Gy will be used for uncomplicated metastases. As there are barely clear recommendations available regarding which radiation schedule to use for “complicated” bone metastases, we will use the available recommendations about spinal bone metastases. Since metastases in the spine often lead to problems of pathological fracture and/or nerve compression, many of these situations are complicated by nature.

For the tumor load, which can be identified as a relevant criterion in the Dutch guideline about spinal metastases, the situation is also complex. Differentiating “oligometastastic” from “polymestatic” cancer situations is a controversial concept without a clear distinction [46]. As there are different and heterogenous definitions of the term “oligometastatic disease”, an ASTRO-ESTRO consensus document has been published to answer some of the key questions regarding the topic [47]. Since there currently is no such thing as a biomarker for the identification of oligometastatic situations, the absolute number of diagnosed metastases is often used for the classification. As a result, oligometastatic diseases are often defined as situations with less than 3 or less than 5 metastases. For our purpose we will be using the same approach, discriminating oligometastatic situations with 1–3 metastases from polymetastatic diseases with > 3 metastases. Yet, one has to be aware that this is a considerable simplification.

Regarding life expectancy, we are also going to use other parameters as surrogates. Several tools and scoring systems exist for the survival prediction of cancer patients with bone metastases. One of these is the model of Bollen et al. who investigated which criteria are significantly associated with median overall survival in patients with spinal bone metastases [48]. In an analysis by Bollen et al. their model performed best among six different prognostic scoring systems for spinal bone metastases [49].

The relevant criteria of this model are primary tumor type [classified into three clinical profiles (Favorable, Moderate and Unfavorable)], Karnofsky Performance Status (KPS) and the presence/absence of visceral and/or brain metastases.

In conclusion, we can identify the following criteria that are both relevant and clearly determinable for the creation of the dose-prescription algorithm (Fig. 4):

- Association of metastasis with pathological fracture
- Association of metastasis with nerve compression of cord or cauda equine
- Number of bone metastases
- Clinical profile (histology of primary tumor)
- KPS
- Presence of visceral/brain metastases

### Step 5: Creation of the clinical algorithm

After the decision criteria and the decisional strategy have been defined, one can create a first draft for a clinical algorithm (analogous to step 5 of the method described by Horabin and Lewis [25].

**Fig. 5:**
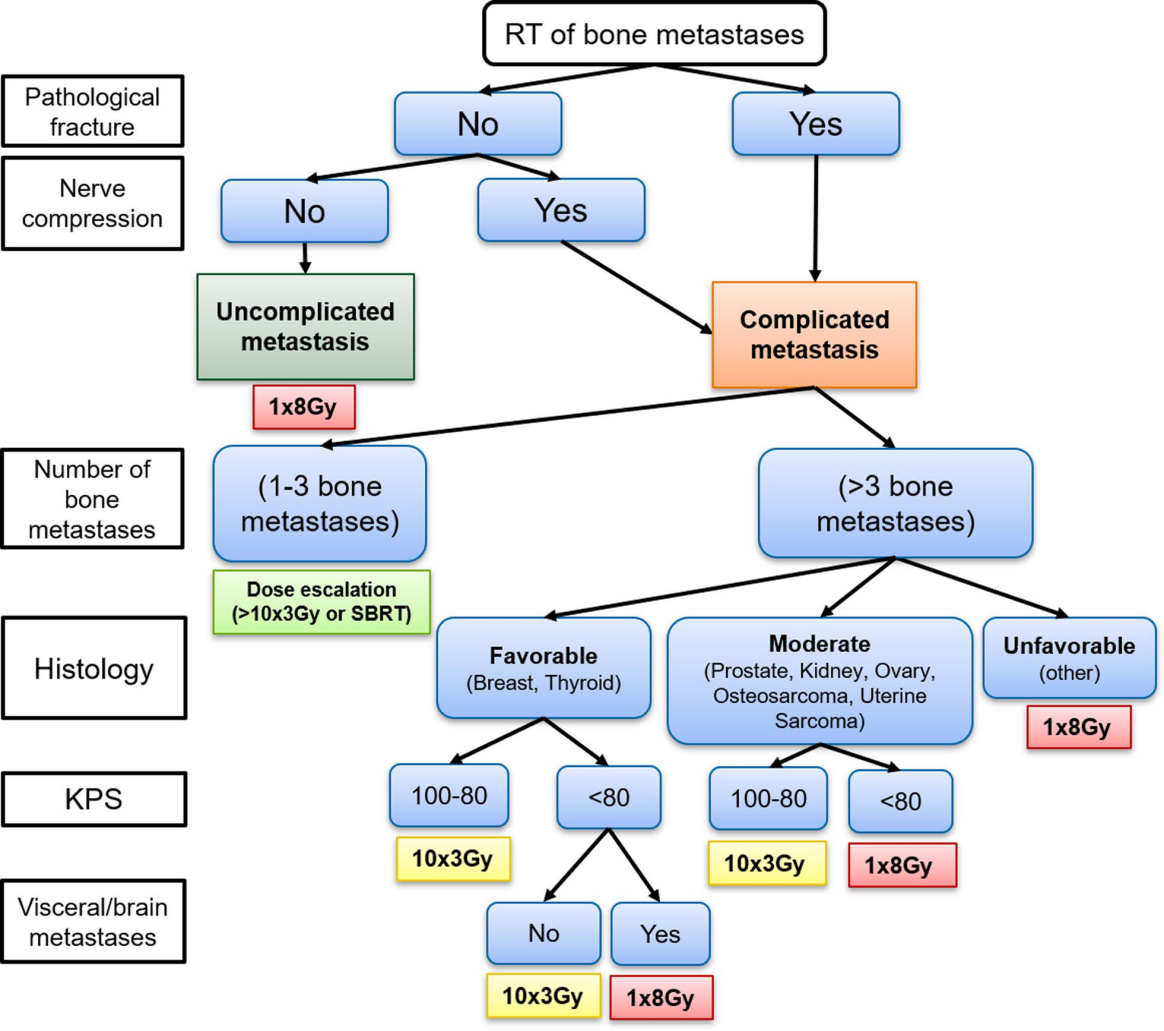
Possible clinical algorithm for dose prescription in palliative radiotherapy of bone metastases

Summarizing the aforementioned consideration, a first draft of a clinical algorithm for dose selection in palliative radiotherapy of bone metastases is demonstrated in (Fig. 5):

Even though, at first glance the algorithm may appear complex, the underlying concept is quite simple. It is basically a structured approach for a possible fractionation scheme based on recommendations given by the ASTRO guideline and the Dutch guideline. First, it is assessed if a metastasis can be classified as complicated. In this case, it is further determined whether a MF scheme or a SF of 1 × 8 Gy should be used depending on the number of overall bone metastases and the estimated life expectancy. Based on the Bollen model and the Dutch guideline, patients with complicated metastases, a polymetastatic situation and a life expectancy of < 6 months will receive a dose of 1 × 8 Gy, while patients with a prognosis of > 6 months will receive 10 × 3 Gy. For patients with a very limited number of ≤ 3 bone metastases it is recommended to further increase the applied dose [e.g. 13 × 3 Gy or stereotactic beam radiotherapy (SBRT)]. Running through the algorithm leads to a fractionation scheme recommended for each individual situation. Other fractionation schemes like 5 × 4 Gy, are commonly used in clinical practice [39]. Though, since there is no such recommendation in the guidelines our algorithm is based on, such an option is not implemented.

### Step 6: Implementation in clinical practice: limitations of structured decision-making and possible solutions

Clinical algorithms for decision-making in oncology may support clinicians and reduce unwarranted practice variation—a known factor to impair patient care and outcome [50]. Yet, there are considerable limitations. To create a clinical algorithm that is useful in daily routine, it is important to be aware of these problems and limitations in order to find solutions for the implementation.

### Individuality of cases → expansion of algorithm

Even in a clearly defined framework like the choice of radiation dose, a clinical algorithm cannot cover all individual clinical situations. Since special cases require flexible management, generalization and “one-size-fits-all”-solutions are not feasible. With the evolving complexity of medical knowledge and the trend towards more personalized patientcare, flexibility becomes even more important. A major problem is that high-level evidence which clearly shows superiority of a specific treatment option, is often missing in specific circumstances. Furthermore, the decision-making takes place in a setting of uncertainties and incomplete information. For individual cases, criteria not included in the algorithm may be considered in decision-making. A possible solution would be the expansion of the algorithm, involving more criteria. To cover as many situations as possible, an algorithm may become very complex. For example, our algorithm does not consider previous irradiation. This is an important factor, as re-irradiation is a common situation for bone metastases and may require adaption of radiation dose. It would be possible to add further criteria, covering these situations as well. However, this holds the risk of adding often negligible criteria into an algorithm. The decision to include criteria into the decision-making process should ideally be based on medical evidence or widely accepted consensus to be valid.

## Practicability of complex algorithms → IT solutions (e.g. CDSS)

Creating complex decision-making strategies makes them impracticable and limits their usage in daily practice. While decision trees may be feasible for illustration when only a couple of criteria are used, handling of complex algorithms is not practicable this way. Implementing even more complex clinical algorithms into clinical practice may be possible using IT-based solutions—namely the implementation into a Clinical Decision Support System. Different kinds of CDSS in oncology have been shown capable of improving safety and efficiency, when used correctly [51]. A CDSS expert system, that gives recommendations for fractionation schemes based on input parameters (decision criteria), may realize complex algorithmic decision-making in a practicable way. However, vast complexity may hinder its successful implementation. In order to be useful in daily practice, the CDSS should work automatically within the clinical workflow, a feature that has been identified to be crucial for the success of a CDSS [5].

## Algorithms should not be too complex (overfitting)

**Fig. 6:**
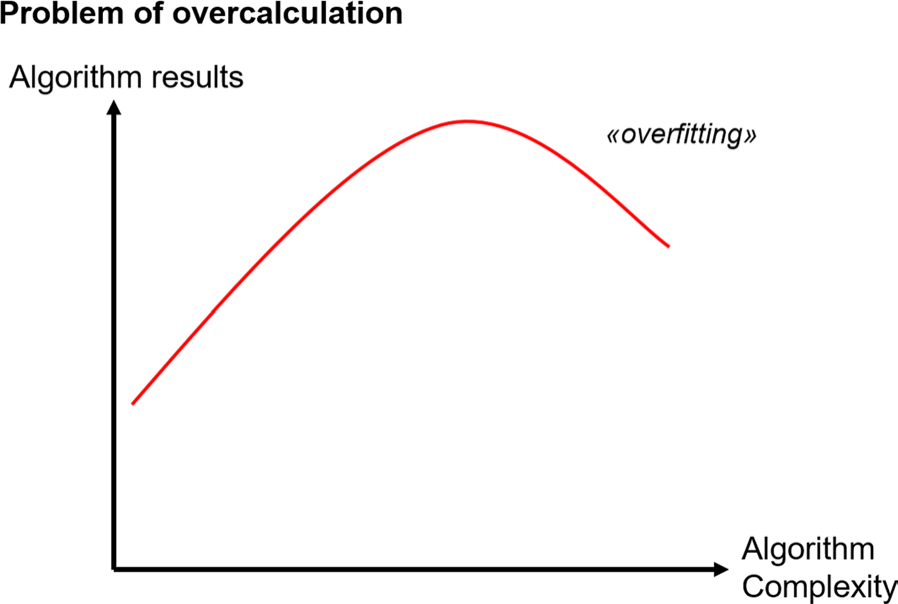
The problem of overfitting

Besides limited applicability of overloaded complex clinical algorithms, there is another reason to keep structured decision-making as simple as possible, which is the problem of overfitting. As the creation of algorithms is based on observations from the past, incorporating as much data as possible is useful for maximal adaption to these past observations. Though that does not mean such a model is useful for predicting the future and making decisions based on that. Every parameter that is measured for decision-making shows some kind of variance or random error. While these parameters or criteria are associated with preference to a certain decision, they are often not directly causative to it. If implemented into a decision strategy, irrelevant or barely relevant criteria may provoke a change of decision solely because of a random error. Rather simple and heuristic strategies outperform too information-greedy algorithms regarding predictive accuracy and decisional outcome [52]. A complex model based on many parameters may be highly accurate regarding past observations—yet the more complex an algorithm becomes, the more prone it is to random parametric errors of this “overfitting” (Fig. 6).

As a result, clinical algorithms should only be as complex as needed and as simple as possible [24]. Not only the question which information to implement as a decision criterion is of importance, but also which information to consciously not include in the decision-making process.

### Step 7: Validation and optimization

After the clinical algorithm has been created and factors regarding clinical implementation have been discussed, it may be used in clinical practice. Yet, there are two important issues that need to be addressed in the post-creation-phase. These are validation and optimization.

## Validation of clinical algorithms in oncology

It is desirable to perform validation and statistical analysis about the quality of the decision recommendations of a clinical algorithm. Yet, particularly in oncology this is not always easy to do. Regarding our example, it is problematic to compare the quality of decisions between the clinical algorithm and the intuitive approach of physicians. Most of the time one cannot tell whether or not one decision is better or worse than another, if ambiguous factors like “avoidance of unnecessary hospital visits” are relevant to the decision. Yet, in oncology, decisions like these are very common. While it would be possible and also interesting to analyze the clinical algorithm regarding other endpoints (e.g. consistency of clinical decision-making), this is beyond the scope of this article.

Obviously, clinical algorithms should be validated wherever possible. But in any case, every recommendation given by an algorithm (validated or not) needs to be evaluated by a physician—algorithms should not replace medical thinking [53].

### Fast development of new therapies → continuous evolvement and adaption of clinical algorithms

Medical science is a rapidly evolving field with growing complexity and improving understanding of underlying mechanisms regarding disease and treatment. A clinical algorithm or any kind of structured decision-making can only lead to optimal decisions when it is based on current medical knowledge. Therefore, continuous evolvement and adaption is required. Regarding palliative irradiation of bone metastases, the usage of stereotactic body radiotherapy is currently investigated and may lead to better results regarding pain relief and local control [54]. There is already some evidence from phase 2 randomized controlled trials indicating improved pain relief of SF SBRT in comparison to MF conventional radiotherapy [55]. As ongoing studies further generate new data, treatment recommendations change and make it necessary to update the algorithm.

Constant adjustment takes a lot of effort—though this is not a problem limited to structured decision-making. Ongoing education and acquisition of the newest medical knowledge is also required for making good decisions within an open and intuitive framework.

### Should the clinical algorithm be designed differently? Data generation and optimization

There remains substantial uncertainty and controversy what fractionation fits an individual patient best. We tried to use guideline recommendations for identifying relevant criteria and deciding which fractionation scheme to choose for palliative irradiation of bone metastases. Yet, our proposed clinical algorithm is not “the one and only” correct way to select a fractionation scheme for an individual oncological case. If the situation was obvious and there was evidence-based data for all possible scenarios, there would be no controversy and it would always be known which dose to choose. Even though the situation is unclear, we still suggest that decisions should be made within a structured framework whenever possible. The clinical algorithm we created is an example of how such an approach might look like.

If decision-making is done within a structured framework, the process itself becomes more transparent and consistent. The decisions are based on the criteria that are considered relevant and are therefore implemented into the algorithm. Due to this, it becomes understandable why a decision was made. If a physician decides to choose another dose than proposed by the clinical algorithm, the deviation from the algorithmic recommendation should be well-founded and documented. Documentation of these reasons is valuable information, that might be used to optimize the clinical algorithm for future use. Generating a structured framework for decision-making thereby also forms the basis for transparent and understandable acquisition of data.

Furthermore, structuring leads to more consistent decision-making and less unwarranted clinical practice variation. Ultimately, it may even enable standardization—mostly within one medical facility, but potentially also on a multicentric level. Efforts for standardization in medicine are desirable as they have been shown to improve clinical safety, quality and efficiency by the prevention of decisional errors [56, 57].

## Conclusion: structured decision-making and clinical algorithms

**Fig. 7:**
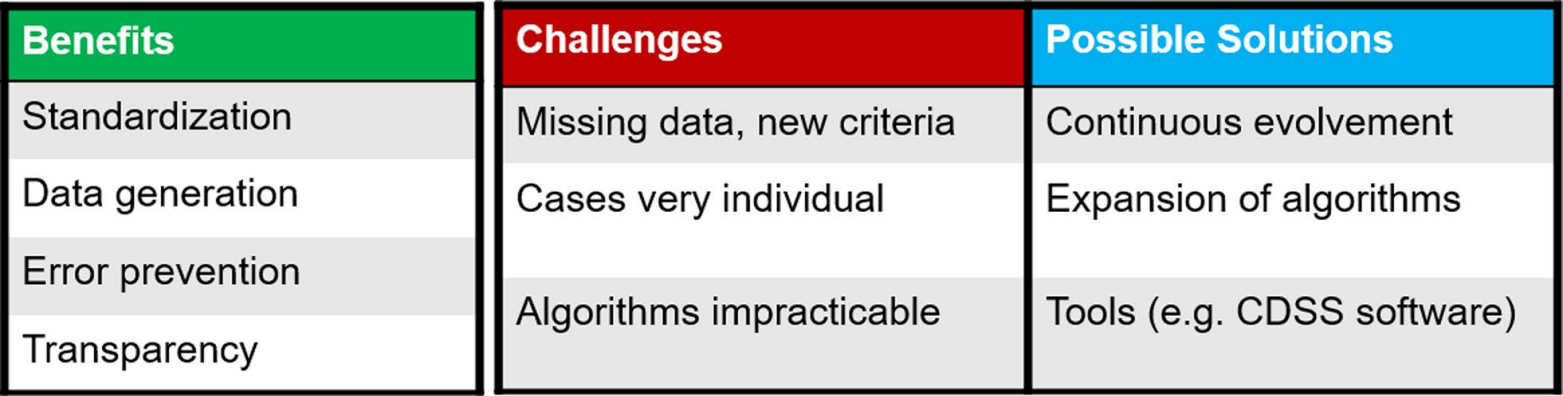
Benefits, challenges and possible solutions of structured decision-making

Structured decision-making in comparison to open-intuitive decision-making may have several advantages. Overall, structuring makes the process of decision-making more comprehensible and transparent. Furthermore, decision theory suggests that structuring can promote system-2-thinking, making the decisional reasoning more conscious and rational. In situations where direct feedback for decision-making is missing (like dose prescription in radiation oncology), the development of good intuitive expertise is limited. Intuitive decision-making tends to overestimate the quality of its judgements and is prone to biases. Structured decision-making may help to overcome some of these issues. Yet there are related challenges for its application for which we offer possible solutions (Fig. 7). As oncological cases are highly individual and medical science is quickly developing, structured decision-making strategies like clinical algorithms need to be constantly adapted and improved to optimally support decision-making. Relevant criteria need to be identified, while barely relevant factors should be out of focus to keep the decision-making practicable and to avoid overfitting. Decision trees as well as IT implementations like CDSS may be helpful tools for the application of decisional algorithms in daily clinical life.

Clinical decision-making in oncology as well as in other medical fields is often complicated, misses consensus and contains uncertainties.

We aimed to demonstrate how a structured framework in the form of a clinical algorithm for supporting decision-making can be created and discuss the necessary steps and factors that need to be considered. We hope that our concept may be a useful orientation for others, who seek a structured approach to medical decision-making in oncology.

## Data Availability

Not applicable.

## Abbreviations

ACLS: Advanced cardiac life support
A.I.: Artificial intelligence
ASTRO: American Society for Radiation Oncology
ATLS: Advanced trauma life support
CDSS: Clinical decision support system
ESTRO: European Society for Radiotherapy and Oncology
KPS: Karnofsky performance status
MF: Multi fractionation
SBRT: Stereotactic body radiotherapy
SF: Single fractionation
SOP: Standard operating procedure
